# dbCRSR: a manually curated database for regulation of cancer radiosensitivity

**DOI:** 10.1093/database/bay049

**Published:** 2018-05-30

**Authors:** Pengbo Wen, Junfeng Xia, Xianbin Cao, Bin Chen, Yinping Tao, Lijun Wu, An Xu, Guoping Zhao

**Affiliations:** 1 Key Laboratory of High Magnetic Field and Ion Beam Physical Biology, Hefei Institutes of Physical Science, Chinese Academy of Sciences, Anhui Province Key Laboratory of Environmental Toxicology and Pollution Control Technology, Hefei, Anhui, People’s Republic of China; 2 University of Science and Technology of China, Hefei, Anhui, People’s Republic of China; 3 Institute of Physical Science and Information Technology, School of Computer Science and Technology, Anhui University, Hefei, Anhui, People’s Republic of China

## Abstract

Radiotherapy is used to treat approximately 50% of all cancer patients, with varying prognoses. Intrinsic radiosensitivity is an important factor underlying the radiotherapeutic efficacy of this precise treatment. During the past decades, great efforts have been made to improve radiotherapy treatment through multiple strategies. However, invaluable data remains buried in the extensive radiotherapy literature, making it difficult to obtain an overall view of the detailed mechanisms leading to radiosensitivity, thus limiting advances in radiotherapy. To address this issue, we collected data from the relevant literature contained in the PubMed database and developed a literature-based database that we term the cancer radiosensitivity regulation factors database (dbCRSR). dbCRSR is a manually curated catalogue of radiosensitivity, containing multiple radiosensitivity regulation factors (395 coding genes, 119 non-coding RNAs and 306 chemical compounds) with appropriate annotation. To illustrate the value of the data we collected, data mining was performed including functional annotation and network analysis. In summary, dbCRSR is the first literature-based database to focus on radiosensitivity and provides a resource to better understand the detailed mechanisms of radiosensitivity. We anticipate dbCRSR will be a useful resource to enrich our knowledge and to promote further study of radiosensitivity.

Database URL: http://bioinfo.ahu.edu.cn:8080/dbCRSR/

## Introduction

Radiotherapy is a commonly used therapeutic modality in cancer treatment, either alone or in combination with other treatment regimens in the clinic (1, 2). This treatment takes advantage of high intensity ionizing radiation to suppress tumour proliferation with no depth restriction and exerts its therapeutic effect mainly via DNA double-strand damage. The optimal goal of radiotherapy is to enhance the tumour radiation response specifically and to reduce toxicity in the surrounding normal tissue (3). However, patients receiving radiotherapy may experience adverse treatment effects due to radioresistance and undifferentiated radiation that is toxic to normal tissue. Acute or early toxicity in normal tissue usually occurs during or within weeks following the treatment. Therefore, developing a precise treatment with appreciable radiosensitivity capacity for cancer tissues is an attractive strategy, which can overcome the undesirable side-effects that impede the use of radiotherapy (4, 5).

Establishing effective treatment methods should be based on comprehensive understanding of the radiosensitivity mechanisms. There has been sustained interest in assessing the biological effects of radiosensitivity due to its potential clinical value (6, 7). Generally, radiosensitivity studies have focused on the molecular mechanisms of radiotherapy with the goal to discover the regulation factors (compounds, genes or gene products) that can enhance cancer radiosensitivity (3, 8). It has been confirmed that DNA repair efficiency, cell cycle arrest, apoptosis and auto-phagy are the main mechanisms associated with radiosensitivity in many cancers (7, 9–11). During the past decades, many radiotherapy regulation factors have been identified. For example, *HIF1A* (hypoxia inducible factor 1 alpha sub-unit) knockouts enhance radiosensitivity by suppressing the DNA double-strand break (DSB) repair pathway (12). Similarly, a decrease in the long non-coding RNA *HOTAIR* (*HOX* transcript anti-sense RNA) efficiently enhances radiosensitivity in breast cancer via the *Akt* pathway (13). Overexpression of *hsa-mir-503* reinforces radiation responses in laryngeal carcinoma (14). Finally, curcumin has been shown to enhance the radiosensitivity of renal cancer cells by suppressing the *NF-κB* signalling pathway (15).

To our knowledge, there is currently no database that specifically focuses on the regulation factors of radiotherapy, although some literature reviews have already summarized several specific mechanisms and listed certain radiosensitivity factors (3). Undigested data buried in the extensive radiotherapy literature may limit the ability to develop a comprehensive understanding of radiosensitivity and impede improvements in radiotherapy. To fill this information gap, we developed a literature-based radiosensitivity regulation factor database (dbCRSR), which is proposed to assist in the investigation of the detailed mechanisms involved in radiotherapy efficacy and improve clinical treatment.

## Materials and methods

### Data collection and database construction

First, to obtain the relevant publications, we performed an extensive PubMed literature query on 9 October 2017, using a list of keywords (Supplementary Table S1). After identifying the appropriate literature, we read through each paper to collect the pertinent information, including publication information, cancer type, radiation ray type and radiosensitivity regulation factors (e.g. genes, miRNAs, lncRNAs and compounds) along with the regulation mode of each factor. Labels ‘up’ or ‘down’ were used to indicate up-regulation or down-regulation of the regulation factors that enhance radiosensitivity. Second, multiple online tools were used to annotate the raw data from PubMed. To enrich the data and to facilitate users, we extracted candidate miRNAs and drugs from the miRTarBase and DrugBank databases, respectively, according to the relevant gene information. The detailed process of data collection can be found in the Supplementary Material (Supplementary Material S1). Finally, to construct the database, we stored and managed all the data in MySQL (version 5.1.73), which is a popular and open-source database management system that has been widely used in biomedical research.

### Data annotation and analysis

MyGene.Info (https://pypi.python.org/pypi/mygene/3.0.0) was used to query gene annotation data. We performed functional analysis using the DAVID (version 6.8) online service (https://david.ncifcrf.gov/) to evaluate the function of the gene set. OmicShare Tools] (www.omicshare.com/tools) were used to analyse the pathway enrichment and to draw the bubble plot. Candidate miRNAs and drugs were annotated from the miRTarBase (http://mirtarbase.mbc.nctu.edu.tw/php/index.php) and DrugBank (https://www.drugbank.ca/) databases, respectively. Statistical calculations were performed using Microsoft Excel 2016. The Venn diagram was drawn by VENNY 2.1 (http://bioinfogp.cnb.csic.es/tools/venny/index.html). The word cloud was created by the Word Cloud Generator (https://wordart.com/create).

## Results

### Data content overview

**Figure 1.**
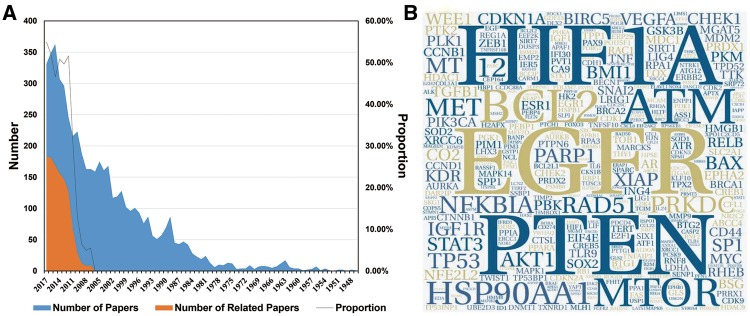
An overview of the data we collected. (**A**) The blue colour indicates the number of papers we gathered from PubMed. The orange colour indicates the number of papers related to the research topic. The black line indicates the proportion of papers (orange/blue) from each year that were relevant to this study. (**B**) The hotspot genes in this study, where larger word size indicates higher frequency of the gene. This format is useful to quickly find the most prominent terms.

A large amount of work has been conducted to study cancer radiosensitivity (Figure 1A). To collect the most relevant data, 1072 of the 2862 papers that were obtained from PubMed and were published in the last decade were extracted for information by manual reading (retrieval formula can be obtained from Supplementary Table S1). In the current version (last updated on 2018-01-01), dbCRSR contains 1132 cancer-sensitivity association entries and includes 54 cancer types, 395 coding genes, 119 non-coding RNAs and 306 compounds. In addition, to visualize the data intuitively, a word cloud was used to exhibit the most-studied genes in this field. As shown in Figure 1B, the epidermal growth factor receptor (*EGFR*), *HIF1A* and phosphatase and tensin homologue (*PTEN*) are the top three genes due to their high frequency. In addition, *has-mir-145*, *has-mir-21* and *has-mir-200c* are the most studied miRNAs in this field (Supplementary Figure S2). Curcumin is the most important drug as it causes multiple cancers to be sensitive to radiation (Supplementary Figures S3–S4).

### Web interface of dbCRSR

**Figure 2.**
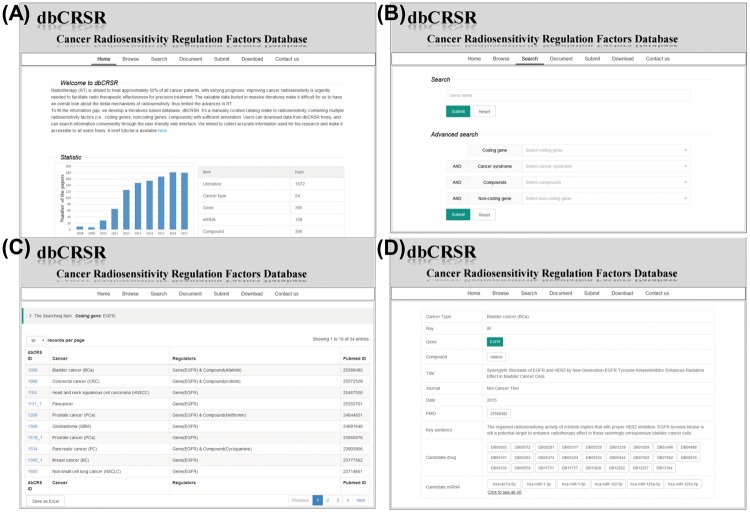
dbCRSR website screen shots illustrating the general search process. (**A**) The dbCRSR home page. (**B**–**D**) A step-by-step example of the searching process.

To enable access to the radiosensitivity data gathered in this study, we developed a dedicated online database, dbCRSR, freely available at http://bioinfo.ahu.edu.cn:8080/dbCRSR/. It contains seven main pages: (i) Home page, which provides an overview of the database (Figure 2A); (ii) Browse page, where users can obtain lists and a summary of the radiosensitivity regulation factors as well as hyperlinks to the detailed evidence and annotation pages; (iii) Search page, where both quick and advanced search options are available depending on the user’s requirement; (iv) Document page, which includes a detailed description of the database; (v) Submit page, where users are encouraged to submit novel data to the database; (vi) Download page, where in addition to customized downloads that are available on the search results page (Figure 2C), bulk download is also enabled for the regulation factors with their annotations and (vii) Contact page, which provides effective contact information. An example (*EGFR*) is given to demonstrate the general process for data searching (Figure 2).

### DNA damage repair-associated pathways are essential for radiosensitivity

**Figure 3.**
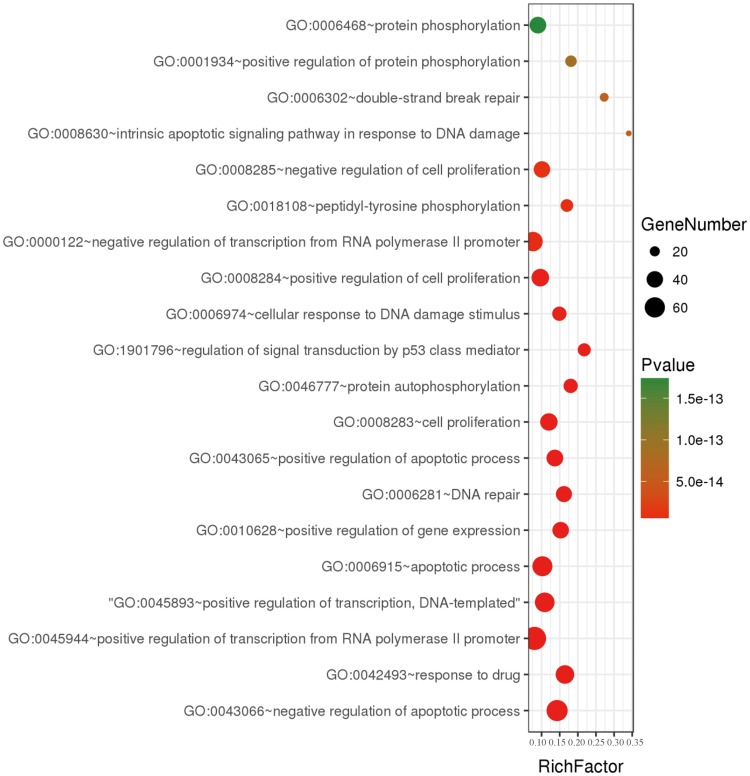
A bubble plot displaying the 20 most significant terms after performing GO enrichment analysis. Bubble colours represent the corrected *P*-value. Bubble sizes indicate the number of genes.

To further understand the features of all collected genes in dbCRSR, we performed gene ontology (GO) enrichment analysis on 395 genes using DAVID (version 6.8). We collected the enriched GO terms with an adjusted *P*-value of <0.05 that was calculated using a hypergeometric test followed by the Benjamini–Hochberg correction. Then, the online OmicShare tools were used to integrate non-redundant GO terms and visualize the significant GO terms (Figure 3, Supplementary Figure S5). We found that most of GO terms were related to DNA damage, double strand breaks and *p53* mediated signal transduction, all of which play important roles in the response to radiation of cancer.

### Down-regulation is the main strategy for radiosensitivity-associated oncogenes but not for tumour suppressor gene

**Figure 4.**
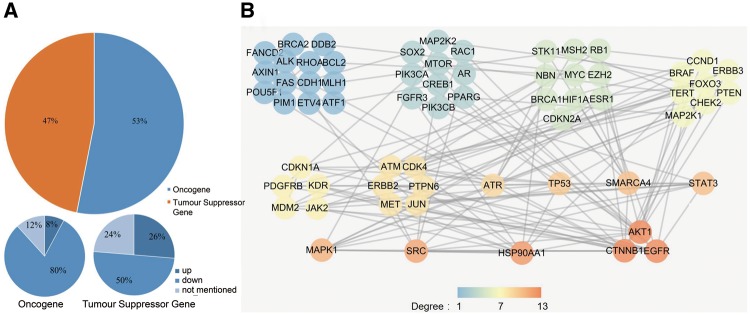
(**A**) The cancer-related genes that were classified as oncogenes and TSGs. (**B**) The PPI network of the collected cancer genes. All genes were sorted by their degree in the network, and all genes with the same degree were clustered together. The degree represents the prominence of the node, which is equal to the number of edges connected to it. The node colour means each gene’s degree.

As previous studies lacked comprehensive classification of the radiosensitivity-related genes, we used the Cancer Gene Census (CGC) (16) to define the genes that we collected as oncogenes or as tumour suppressor genes (TSGs) according to their function during tumourigenesis. We found that 82 of the 395 dbCRSR genes were also present in CGC. After determining the mode of regulation for the 82 genes (53% oncogene and 47% TSG, Figure 4A), we found that decreasing the activity of the oncogenes (down-regulation) was the main strategy that contributed to radiosensitivity. In contrast, there was no specific regulation trend for TSGs. To further analyse these 82 genes, we performed protein-protein interaction (PPI) analysis by using an online tool, STRING (Figure 4B). To describe the sub-networks, we also analysed the topological properties of genes that were correlated with each other using Cytoscape (version 3.5.1). The degree represents the number of edges adjacent to a node, which is one of the most noteworthy features in the PPI network. From this analysis, we found that *EGFR*, catenin beta 1 (*CTNNB1*)*, AKT* serine/threonine kinase 1 (*AKT1*), heat shock protein 90 alpha family class A member 1 (*HSP90AA1*)*, SRC* proto-oncogene non-receptor tyrosine kinase (*SRC*) and mitogen-activated protein kinase 1 (*MAPK1*) were the genes with the highest degree (Figure 4B), indicating their important roles in radiosensitivity.

### Five genes were selected after considering their broad-spectrum radiosensitivity function and the network degree

**Figure 5.**
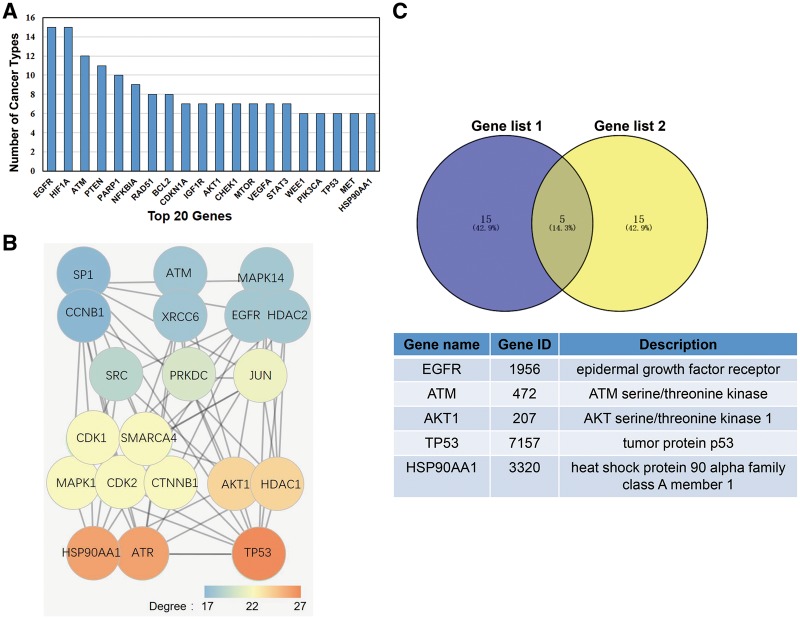
(**A**) The top 20 genes that function in multiple cancers. (**B**) The top 20 genes with the highest degree in the PPI network that were selected from the 395 genes included in this database. The colour indicates the degree of each gene. (**C**) Five genes were selected after overlaying the two gene sets.

In this study, we deemed a gene as having broad spectrum radiosensitivity if it induces radiotherapy sensitization in multiple cancers. To determine which genes induced broad spectrum radiosensitivity, we calculated the number of different cancer types that were susceptible to radiotherapy for each gene in dbCRSR. The top 20 genes that induced radiosensitivity in the most cancer types are listed in Figure 5A. Previous studies have demonstrated that in the PPI network, nodes with higher degrees tend to be important (17); therefore, we created another list of top 20 genes due to their higher degree in the PPI network (Figure 5B). To determine the most important genes from the 395 genes included in this database, we overlaid the two gene sets and five genes stood out (Figure 5C).

## Discussion

Elucidating the mechanisms involved in radiosensitivity is essential to optimize tumour cell susceptibility to radiation while simultaneously minimizing radiotoxicity in normal tissues. Previous studies typically evaluated only a limited number of related genes and therefore failed to draw any comprehensive conclusions. In this study, numerous regulation factors that increase the radiosensitivity of cancer cells were collected in our database. Moreover, all regulation factors along with their associated annotation and analysis results are freely downloadable for further scientific research.

Due to clear clinical significance of radiotherapy, numerous studies have been carried out to investigate the effect of radiotherapy of cancer as well as the mechanisms responsible for cancer radiosensitivity. As expected, the most studied genes in this field (Figure 1B) are also important genes that are essential for other pivotal bioprocesses. For example, deficient signalling of the *EGFR* and other receptor tyrosine kinases is associated with human diseases such as Alzheimer’s, while *EGFR* overexpression is associated with the development of a variety of tumours (18, 19). The act of regulating *HIF1A* can either turn off or turn on processes critical for mammalian regeneration (20). *PTEN* is one of the most commonly lost TSGs in human cancer (21). Studying the role of these important genes in response to radiation can help to explain their capability for enhancing radiosensitivity.

Previous studies have shown cancer radiosensitivity to be primarily related to DNA damage repair, oxidative stress and apoptosis pathways. To verify whether the genes collected in dbCRSR were involved in these pathway, GO term enrichment was performed to interpret the function of the 395 genes in the database. The results from GO enrichment analysis (Figure 3 and Supplementary Figure S5) are consistent with those from previous studies (3–5, 22), which further confirms that DNA damage repair, oxidative stress and apoptosis are the main processes related to radiation response. Therefore, suppressing the DSB repair pathway can enhance ionizing radiation-induced cellular damage during radiotherapy.

Oncogenes promote cell proliferation and differentiation; thus, reducing the expression level of oncogenes in cancer could negatively regulate tumourigenesis. In this study, we found decreased oncogene activity to be the major regulation strategy (80%) for radiosensitivity (Figure 4A). In addition, the three highest-degree genes (*EGFR, CTNNB1, AKT1*) in the PPI network are all known oncogenes, and down-regulation of these three genes can enhance radiosensitivity (Figure 4B). In contrast, we found no specific trend regarding TSGs and radiosensitivity.

To further demonstrate the value of the data we collected, we performed the following analysis. The 20 genes listed in Figure 5A reflect their universal function in radiotherapy across different cancer types. The 20 genes with higher degree in the PPI network shown in Figure 5B reflect their tight interaction with other genes. After taking the intersection of these two gene sets, five top genes were selected. According to previous studies, all five genes play crucial roles in radiation response. For example, (i) radiation combined with *EGFR* blockade inhibited tumour proliferation, increased apoptosis, prolonged G2/M arrest and significantly enhanced DNA injury in colorectal cancer (23). (ii) Inhibition of the phosphorylation of *ATM* could slow DSB repair kinetics (24). (iii) In human oesophageal squamous cell carcinoma cells, AKT phosphorylation contributes to tumour aggressiveness, invasiveness and resistance to radiation therapy (25). (iv) In non-small cell lung cancer cells, *p53* regulates radiosensitivity by inhibiting auto-phagy and activating apoptosis (26). (v) Combining *HSP90* inhibitors with low-dose ionizing radiation enhances the radiosensitivity of HCT116 cells (27).

In this study, we constructed a literature-based resource for radiosensitivity and used several analyses to display the data we collected. However, some limitations remain. First, because of the lack of radiotherapy-related data, the current PPI network was acquired from STRING, which is based on publications. Therefore, if the genes/proteins are frequently studied, more data will support the PPI network, which can generate a biased network. As a result, with the radiotherapy-related expression data increasing in the future, other network such as co-expression network can be used to investigate the specific cancer. Second, because of genetic differences between individuals, patients’ responses to preoperative radiotherapy are distinct. Thus, it is essential for future studies to identify specific radiotherapy biomarkers. For example, Zhu *et al*. have identified effective miRNA biomarkers for predicting the response of colorectal cancer to chemoradiotherapy (27).

In conclusion, dbCRSR represents the first attempt to establish a literature-based resource for cancer radiosensitivity. It is a resource for better understanding the detailed mechanisms responsible for radiation response and for developing an effective strategy to accelerate the progress of personalized medicine in radiotherapy. We expect that dbCRSR will be a valuable resource for precision medicine in cancer radiotherapy.

## Supplementary data

Supplementary data are available at *Database* Online.

## Funding

This work was supported by CAS Pioneer Hundred Talents Program (GZ), National Natural Science Foundation of China (31601124, 61672037 and 31470829), the Anhui Provincial Natural Science Foundation (1708085MC66), the Anhui Provincial Outstanding Young Talent Support Plan (gxyqZD2017005) and the Young Wanjiang Scholar Program of Anhui Province, China.

*Conflict of interest*. None declared.

## Supplementary Material

bay049_supp
