# Supplementary material for: dbCRSR: a manually curated database for regulation of cancer radiosensitivity
Source: Database (Oxford). 2018 May 30;2018:bay049. doi: 10.1093/database/bay049 (PMC6007213; doi:10.1093/database/bay049)
Supplement: bay049_supp [file bay049_supp.doc]

**Supplementary materials**

- **Data collection and annotation**

Supplementary Table S1. Search strategy

Supplementary Figure S1. Work flow of data collection and annotation

- **Statistics of the data**

Supplementary Figure S2. 10 highest frequency miRNAs.

Supplementary Figure S3. 10 highest frequency compounds.

Supplementary Figure S4. Top 10 compounds function in multiple cancers.

- **Analysis of the data**

Supplementary Figure S5. The results of GO analysis.

| **Recent queries in pubmed** | | |
| --- | --- | --- |
| Search | Query | Items found |
| **#6** | **Search (#1) AND #2** | **5686** |
| #2 | Search ((radiosensitivity[Title/Abstract]) OR radio-sensitivity[Title/Abstract]) OR radiation sensitivity[Title/Abstract] | 11438 |
| #1 | Search ((((tumor[Title/Abstract]) OR cancer[Title/Abstract]) OR carcinoma[Title/Abstract]) OR blastoma[Title/Abstract]) OR neoplasm[Title/Abstract] | 2258600 |

Supplementary Table S1. Search strategy


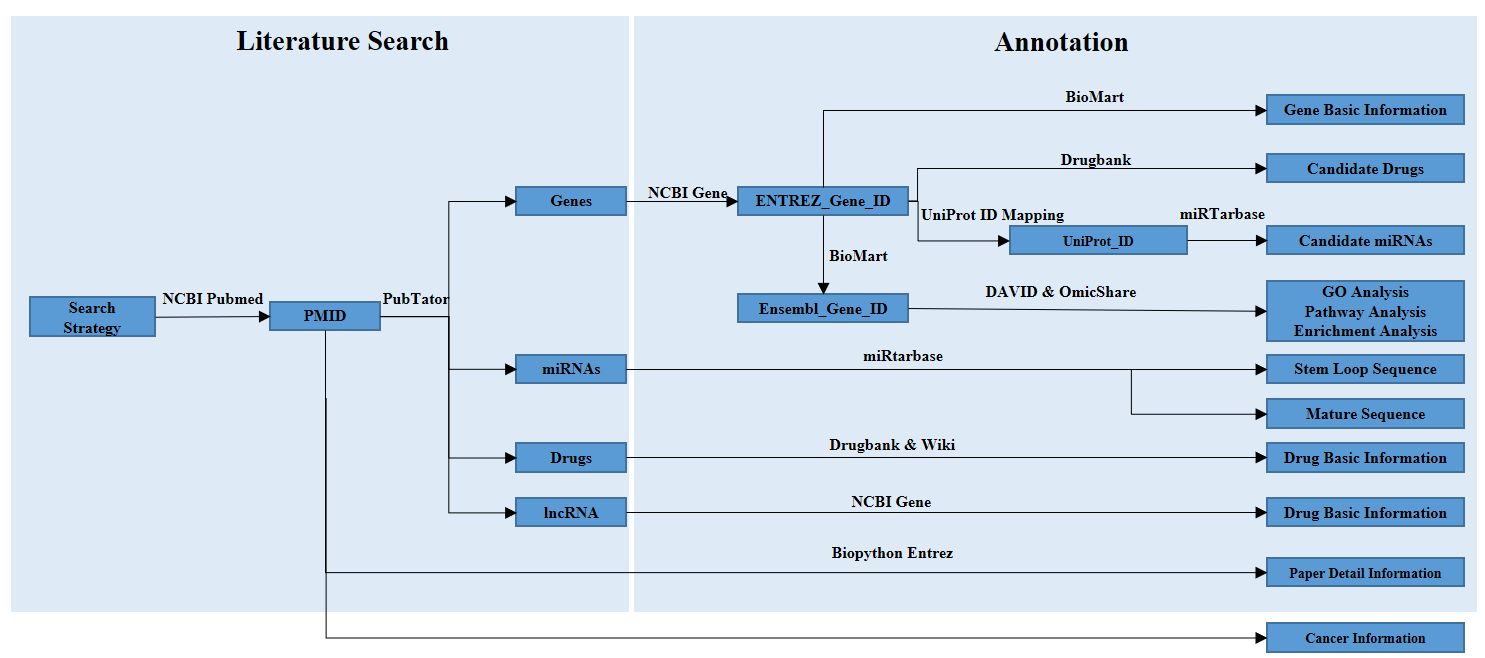


Supplementary Figure S1. Work flow of data collection and annotation


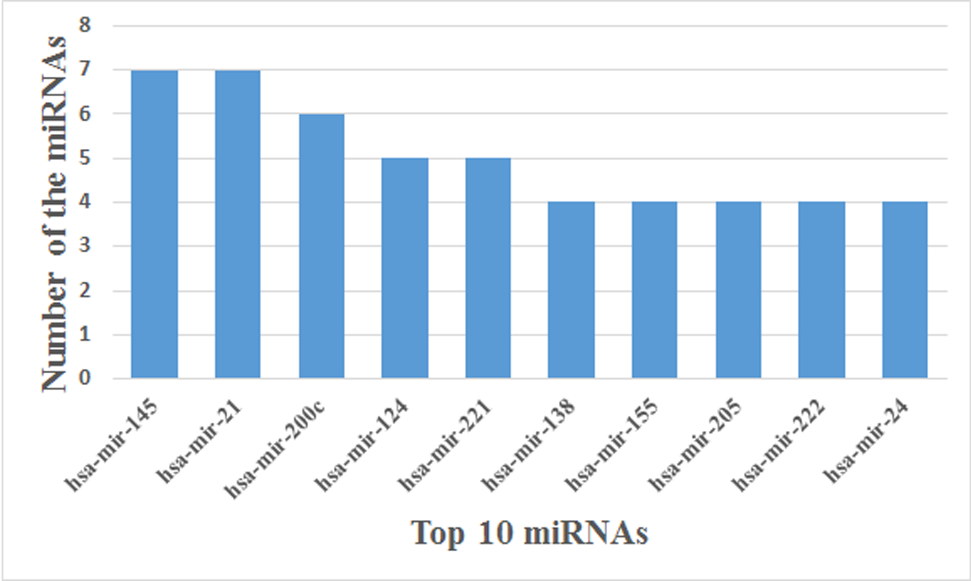


Supplementary Figure S2. 10 highest frequency miRNAs.


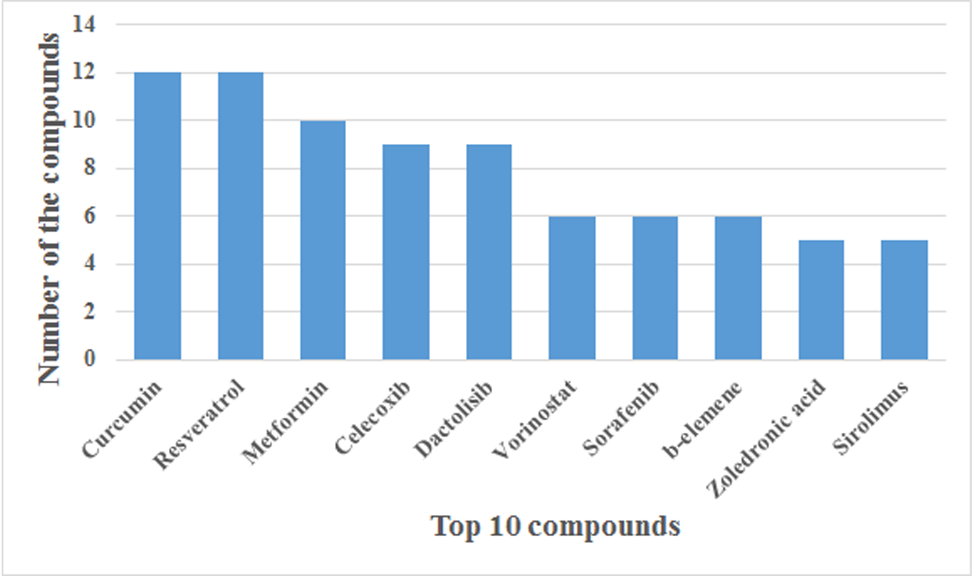


Supplementary Figure S3. 10 highest frequency compounds.


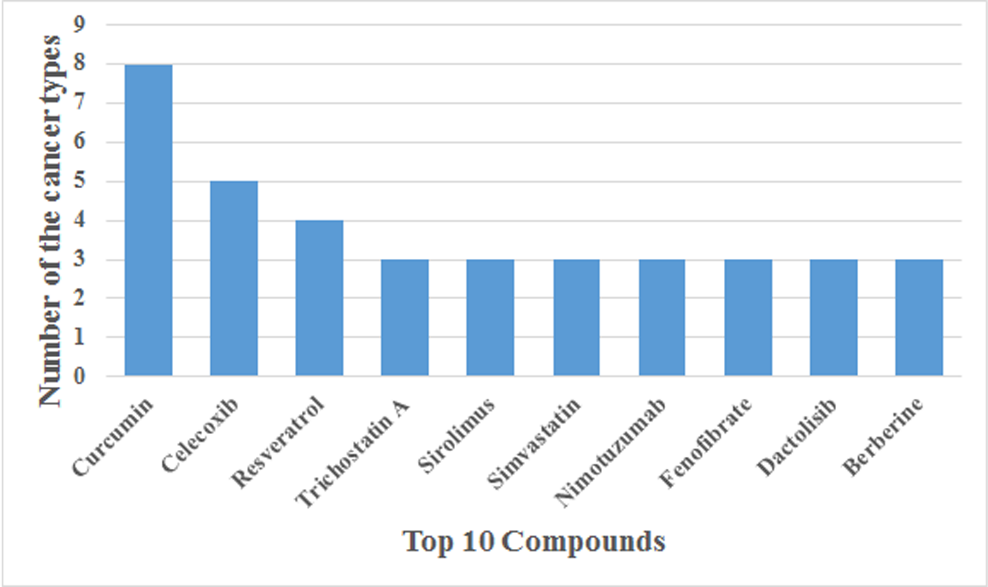


Supplementary Figure S4. Top 10 compounds function in multiple cancers.


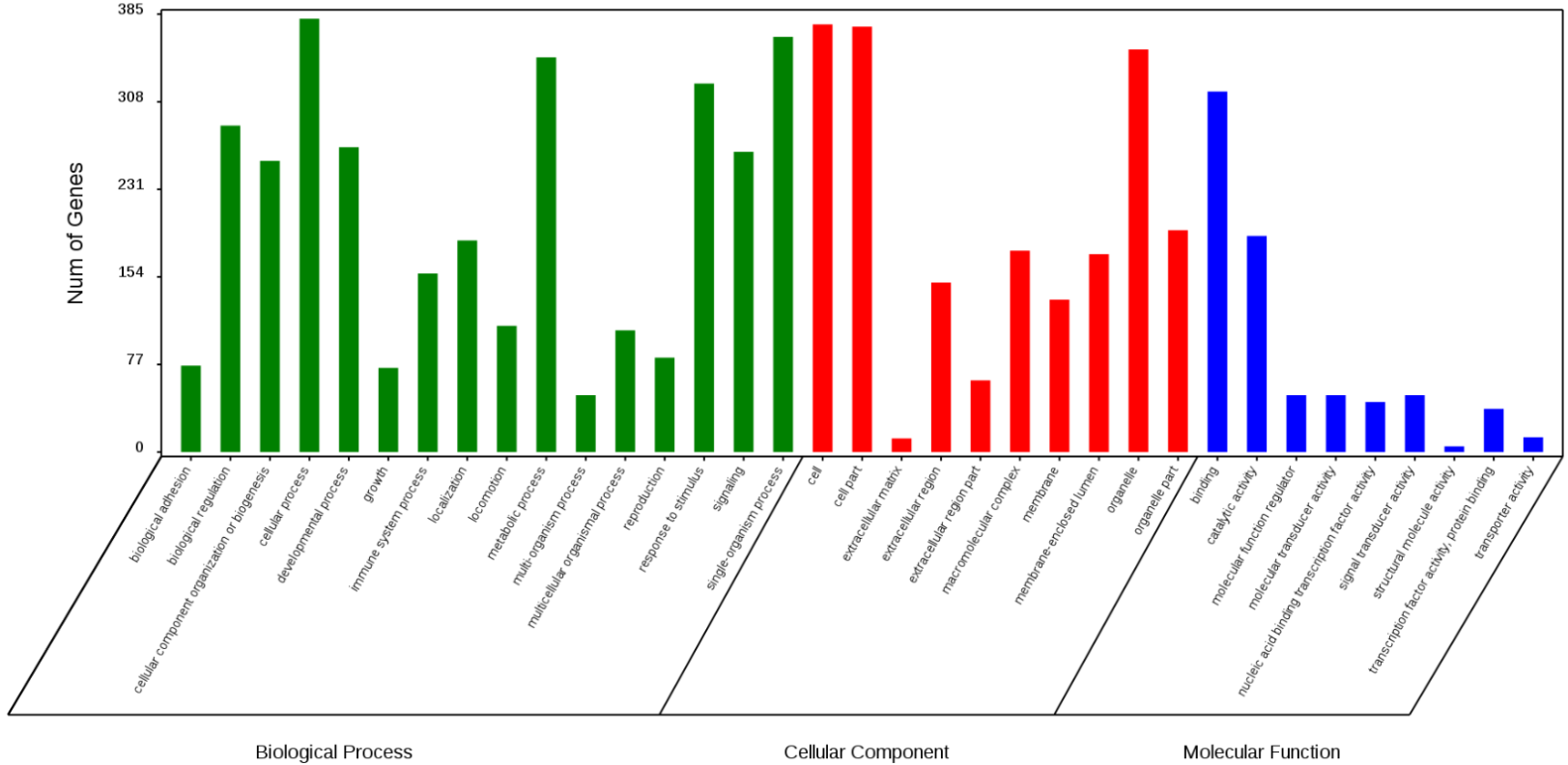


Supplementary Figure S5 GO enrichment analysis
